# Twinfilin-2a Is Dispensable for Mouse Development

**DOI:** 10.1371/journal.pone.0022894

**Published:** 2011-08-18

**Authors:** Elisa M. Nevalainen, Attila Braun, Maria K. Vartiainen, Martina Serlachius, Leif C. Andersson, Markus Moser, Pekka Lappalainen

**Affiliations:** 1 Institute of Biotechnology, University of Helsinki, Helsinki, Finland; 2 Department of Veterinary Biosciences, University of Helsinki, Helsinki, Finland; 3 Max Planck Institute of Biochemistry, Department of Molecular Medicine, Martinsried, Germany; 4 HUSLAB, Department of Pathology, University of Helsinki, Helsinki, Finland; Cardiff University, United Kingdom

## Abstract

Twinfilins are evolutionarily conserved regulators of cytoskeletal dynamics. They inhibit actin polymerization by binding both actin monomers and filament barbed ends. Inactivation of the single *twinfilin* gene from budding yeast and fruit fly results in defects in endocytosis, cell migration, and organization of the cortical actin filament structures. Mammals express three twinfilin isoforms, of which twinfilin-1 and twinfilin-2a display largely overlapping expression patterns in non-muscle tissues of developing and adult mice. The expression of twinfilin-2b, which is generated through alternative promoter usage of the *twinfilin-2* gene, is restricted to heart and skeletal muscles. However, the physiological functions of mammalian twinfilins have not been reported. As a first step towards understanding the function of twinfilin in vertebrates, we generated twinfilin-2a deficient mice by deleting exon 1 of the *twinfilin-2* gene. Twinfilin-2a knockout mice developed normally to adulthood, were fertile, and did not display obvious morphological or behavioural abnormalities. Tissue anatomy and morphology in twinfilin-2a deficient mice was similar to that of wild-type littermates. These data suggest that twinfilin-2a plays a redundant role in cytoskeletal dynamics with the biochemically similar twinfilin-1, which is typically co-expressed in same tissues with twinfilin-2a.

## Introduction

The actin cytoskeleton plays an essential role in several cell biological processes, such as cell motility, morphology, endocytosis, and cell division [1]–[3]. In cells, actin dynamics are tightly regulated by a large number of actin-binding proteins. Twinfilins are conserved proteins, which regulate actin dynamics by sequestering actin monomers, severing actin filaments, and capping actin filament barbed ends. These approximately 40 kDa proteins consist of two actin-depolymerizing factor homology (ADF-H) domains, a linker region, and a C-terminal tail [4]–[14]. The N-terminal ADF-H domain of twinfilin binds only monomeric actin, whereas the C-terminal ADF-H domain also displays weak actin filament binding activity [8], [9]. Although both ADF-H domains separately bind actin monomers, the presence of two functional ADF-H domains is needed for actin filament barbed end capping activity [6]–[8].

In addition to actin, twinfilins interact with the heterodimeric capping protein and PI(4,5)P_2_ [10]–[12]. At least in budding yeast, the interaction with capping protein is essential for the correct sub-cellular localization of twinfilin. This interaction does not, however, affect the actin-binding activities of either protein [10], [13]. The interaction with PI(4,5)P_2_ down-regulates the actin binding of twinfilin [10], [11], [14].

Twinfilin, first identified in yeast, has subsequently been identified in all eukaryotes studied, except in plants [4], [12], [15]. In lower eukaryotes typically one twinfilin protein is present, while mammals (at least rodents and primates) express three distinct isoforms from two twinfilin genes (*Twf1* and *Twf2*). The *Twf2* gene encodes for two different Twf2 isoforms (Twf2a and Twf2b), which are generated by alternative promoter usage. Consequently, the two Twf2 variants differ only in the very N-terminal region and are identical with each other from Twf2a residue 9 and Twf2b residue 7 onwards [11], [12]. All three isoforms bind actin monomers, filament barbed ends, and heterodimeric capping protein, but display small differences in their affinities for monomeric actin [12]. In mice, twinfilin-1 is the most abundant isoform during development as well as in most adult mouse non-muscle tissues. Twinfilin-2a expression increases during development and in adult mouse tissues this isoform is typically co-expressed in same tissues and cell-types with twinfilin-1. Twinfilin-2b, which is generated from the same gene as twinfilin-2a through alternative promote usage, is expressed exclusively in heart and skeletal muscles [11], [12].

Deletion of the *twinfilin* gene from budding yeast results in defects in the organization of cortical actin patches. Together with certain *cofilin* and *profilin* mutations, *twinfilin* deletion leads to synthetic lethality [4]. Inactivation of *twinfilin* in flies led to severe defects in a number of actin-dependent processes. These include e.g. abnormal bristle and eye morphology as well as defects in axonal growth in the brain and border cell migration in the ovary [16], [17]. Twinfilins also play an important role in actin-dependent processes in cultured mammalian cells, because depletion of either twinfilin-1 or twinfilin-2a from HeLa cells by RNAi resulted in defects in endocytosis and depletion of twinfilin-2a from SH-SY5Y cells restrained neurite outgrowth [18], [19]. Furthermore, depletion of twinfilin-1 results in defects in invasive motility of lymphoma cells, whereas up-regulation of twinfilin-1 promoted cardiac hypertrophy [20], [21]. However, despite the wealth of data on cultured mammalian cells, the role of mammalian twinfilin isoforms in animal models has not been reported.

As a first step towards understanding the role of twinfilins in mammalian development and physiology, we generated twinfilin-2a knockout mice. Surprisingly, although studies on cultured cells indicated that this protein plays an important role in endocytosis and neurite outgrowth [18], [19], ablation of twinfilin-2a did not lead to obvious defects in mouse development. These data suggest that in mice twinfilin-2a and twinfilin-1 may have redundant roles in promoting actin dynamics in non-muscle cells.

## Results

### Generation of twinfilin-2a deficient mice

**Figure 1:**
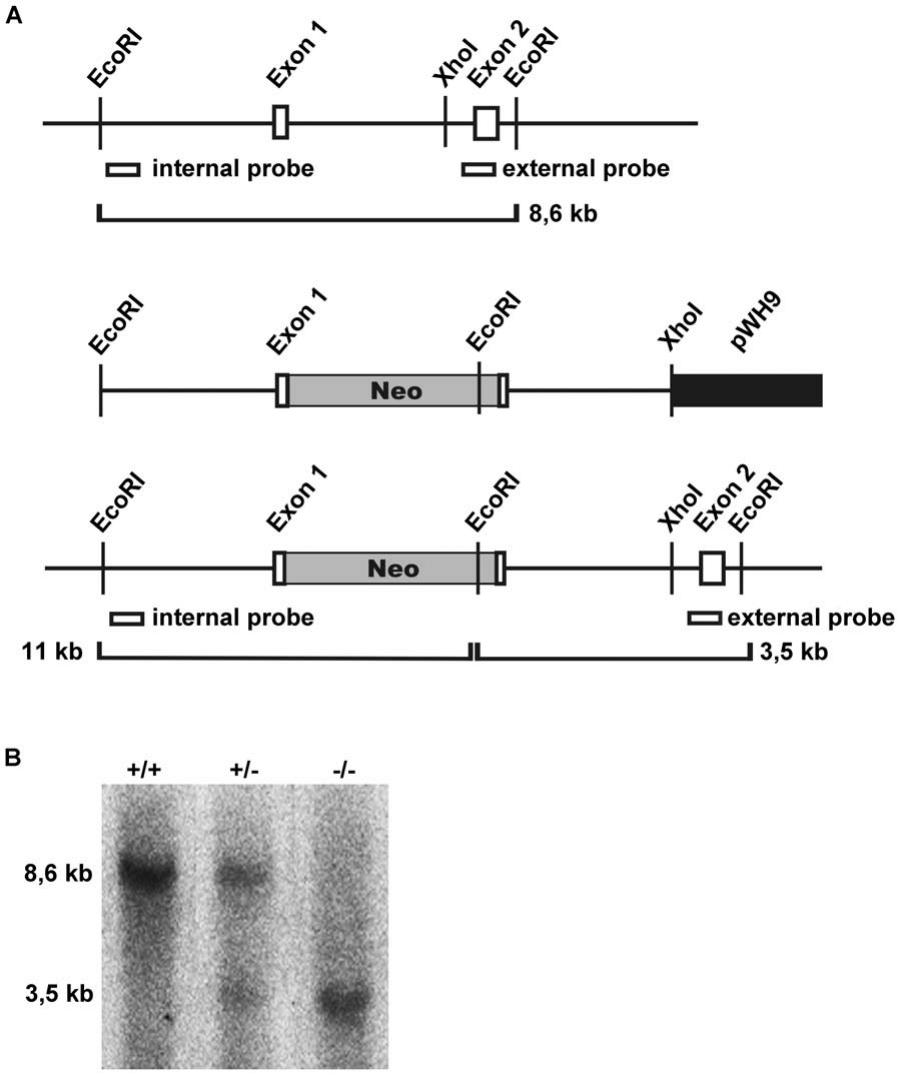
Generation of twinfilin-2a knockout mice. (A) Partial map of the wild-type *Twf2* allele, the targeting construct and the recombinant allele, and locations of the probes. The white boxes show the exons of the *twinfilin-2* gene and the shaded box the neomycin cassette, black box indicates the pWH9 vector. The expected fragment sizes of the wild type and the recombinant allele after digestion with EcoRI and subsequent hybridization with the indicated external probe are 8,6 and 3,5 kb, and with the indicated internal probe, 8,6 and 11 kb, respectively. (B) Southern blot analysis with the external probe of EcoRI digested mouse tail DNAs derived from progeny of heterozygous mating reveals the wild-type (8,6 kb) and the targeted *Twf2* allele (3,5 kb). [+/+ wild type, +/− heterozygote, −/− knockout].

In mice, three twinfilin isoforms are expressed, of which twinfilin-2a and twinfilin-2b are derived from the same gene through alternative promoter usage [12]. The mouse *twinfilin-2* gene consists of nine exons of which the first one is omitted from the twinfilin-2b transcript. To generate twinfilin-2a knockout mice, the genomic fragment containing exon 1 and exon 2 of the *twinfilin-2* gene was isolated from a PAC library. This fragment was used for creating a construct with a neomycin expression cassette (Figure 1A). Exon 1 of the mouse *twinfilin-2* gene, containing the translation initiation codon for twinfilin-2a protein, was inactivated by homologous recombination in embryonic stem (ES) cells. From approximately 220 ES cell clones collected after G418 selection, 5 correctly targeted clones were identified by Southern blot hybridization from EcoRI-digested genomic DNA using an external and an internal probe (data not shown). The knockout strategy and locations of the sites for internal and external probes are presented in Figure 1A.

**Figure 2:**
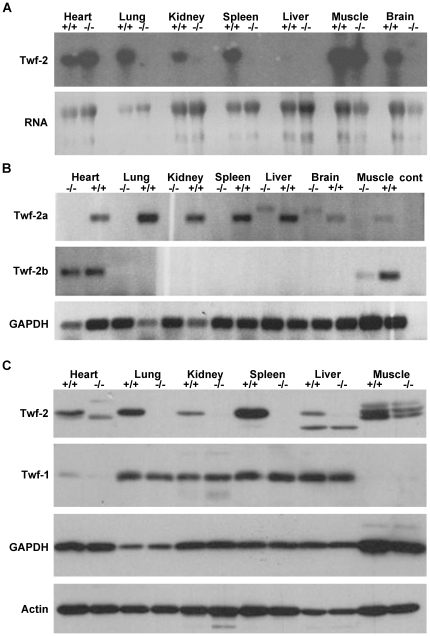
Northern blot, RT-PCR, and Western blot analysis of twinfilin-2a knockout mice. Total RNAs and proteins were isolated from 3-month-old wild-type and twinfilin-2a knockout littermates. (A) Northern blot analysis of the total RNA of wild type (+/+) and knockout (−/−) mice hybridized with a twinfilin-2 cDNA probe. Twinfilin-2 RNA is absent from all mutant tissues, except in heart and skeletal muscles. The remaining twinfilin-2 mRNA encodes for the twinfilin-2b isoform. Equal loading between the wild-type and knockout RNAs is demonstrated in the lower panel. (B) Agarose gel electrophoresis of products from RT-PCR of total tissue RNA of wild type (+/+) and knockout (−/−) mice. Twinfilin-2a mRNA is absent from all mutant tissues, whereas twinfilin-2b expression remains in heart and skeletal muscles of twinfilin-2a knockout mice. Lower panel shows control reactions carried out by using GAPDH-specific primers. The slower mobility band detected in liver and brain of knockout mice was isolated and sequenced and turned out to be an unspecific product of an unrelated protein. (C) Western blot of tissue homogenates of wild type (+/+) and knockout (−/−) mice probed with twinfilin-2 (Twf-2) or twinfilin-1 (Twf-1) specific polyclonal antibodies. Twinfilin-2 protein was absent from all other mouse tissues tested except heart and skeletal muscles. Please note that the twinfilin-2 antibody used here does not distinguish between twinfilin-2a and twinfilin-2b isoforms. GAPDH and actin are shown as loading controls.

ES cells from three targeted clones were used to generate germ line chimeras, which were subsequently mated with C57BL/6 wild type mice to generate heterozygous offspring. For one of the three clones, germ line transmission was achieved. Heterozygous breeding produced *Twf-2a^+/+^*, *Twf-2a^+/−^* and *Twf-2a^−/−^* offspring in normal Mendelian ratio, indicating that twinfilin-2a deficiency does not lead to embryonic lethality (data not shown). The genotypes were confirmed by Southern blot hybridizations with an external probe (Figure 1B) and an internal probe (data not shown) and PCR (data not shown). The absence of twinfilin-2a mRNA was confirmed by Northern blot hybridization (Figure 2A) and RT-PCR (Figure 2B), and the absence of twinfilin-2a protein by Western blot (Figure 2C).

Northern blot analysis of several mouse heart, lung, kidney, spleen, liver, skeletal muscle, and brain extracts revealed that twinfilin-2 mRNA is expressed in all wild-type mouse tissues except liver. In corresponding mutant tissues, however, a twinfilin-2 mRNA transcript remained in heart and skeletal muscle only (Figure 2A). Since the knockout cassette disrupts exon 1 of the *twinfilin-2* gene, the twinfilin-2b transcript is not affected by the knockout construct. These data are in line with the observations that twinfilin-2b, which does not use the exon 1 of mouse *twinfilin-2* gene, is strongly expressed in skeletal muscle and heart [12].

To test for the presence of twinfilin-2a/2b mRNAs in our knockout mice in more detail, we performed RT-PCR with primers that distinguish twinfilin-2a from twinfilin-2b transcripts. RT-PCR analysis revealed that twinfilin-2a is indeed absent from all knockout mouse tissues, whereas twinfilin-2b persists in heart and skeletal muscle, confirming the Northern blot results (Figure 2B). The slower mobility band detected in kidney and brain extracts of the knockout mice was sequenced and turned out to be an unrelated protein (data not shown).

Western blot analysis using isoform-specific antibodies [11] revealed that twinfilin-2 protein was present at detectable levels in all tissues of the analyzed wild-type mouse, whereas twinfilin-2 protein was undetectable in all knockout mouse tissues except heart and skeletal muscle (Figure 2C). It is important to note, that this antibody does not distinguish between twinfilin-2a and twnfilin-2b proteins. In heart extracts, a slightly smaller protein, representing twinfilin-2b, was detected. In skeletal muscle extracts, three proteins with slightly different molecular weights were detected, most likely resulting from post-translational modification of twinfilin-2b. The smaller protein from wild-type and knockout mouse liver extracts is most likely a result of unspecific binding of the twinfilin-2 antibody.

**Figure 3:**
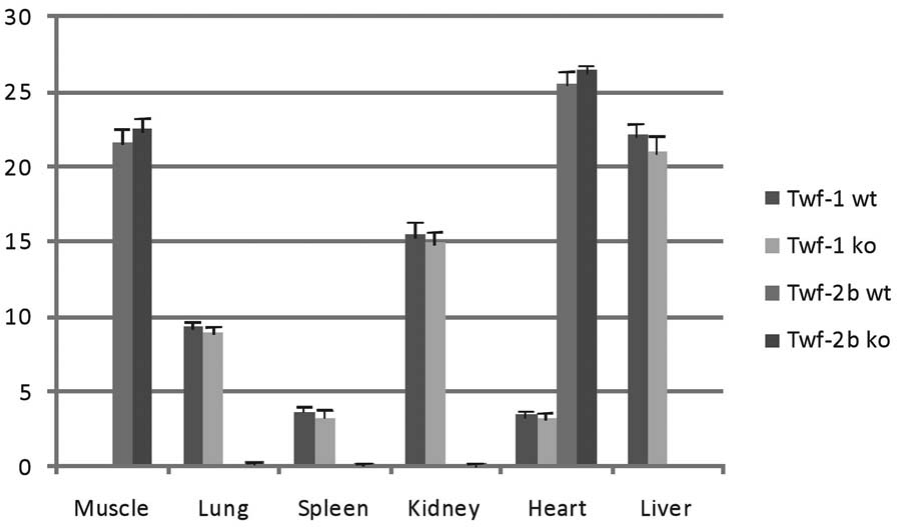
qRT-PCR analysis of twinfilin-1 and twinfilin-2b expression in wild-type and twinfilin-2a knockout tissues. *Gapdh* and *beta-actin* amplification was used as a control. *Twf2b* is the most abundant isoform in heart and skeletal muscles, whereas *Twf1* is widely expressed in non-muscle tissues. The expression levels of either *Twf-1* or *Twf-2b* are not significantly altered in the tissues of *Twf-2a* knockout mouse compared to the wild-type tissues.

The Western blot experiments suggest that twinfilin-1 protein is present in all tissues, with the exception of heart where it is expressed only weakly and skeletal muscle where it is absent. The levels of twinfilin-1 protein do not seem to change significantly in twinfilin-2a knockout mouse tissues (Figure 2C). Furthermore, qRT-PCR experiments did not reveal significant changes in the twinfilin-1 or twinfilin-2b mRNA expression levels in twinfilin-2a knockout mice, suggesting that depletion of twinfilin-2a is not compensated by upregulation of the two other isoforms (Figure 3).

### Twinfilin-2a deficient mice do not display gross developmental defects

**Figure 4:**
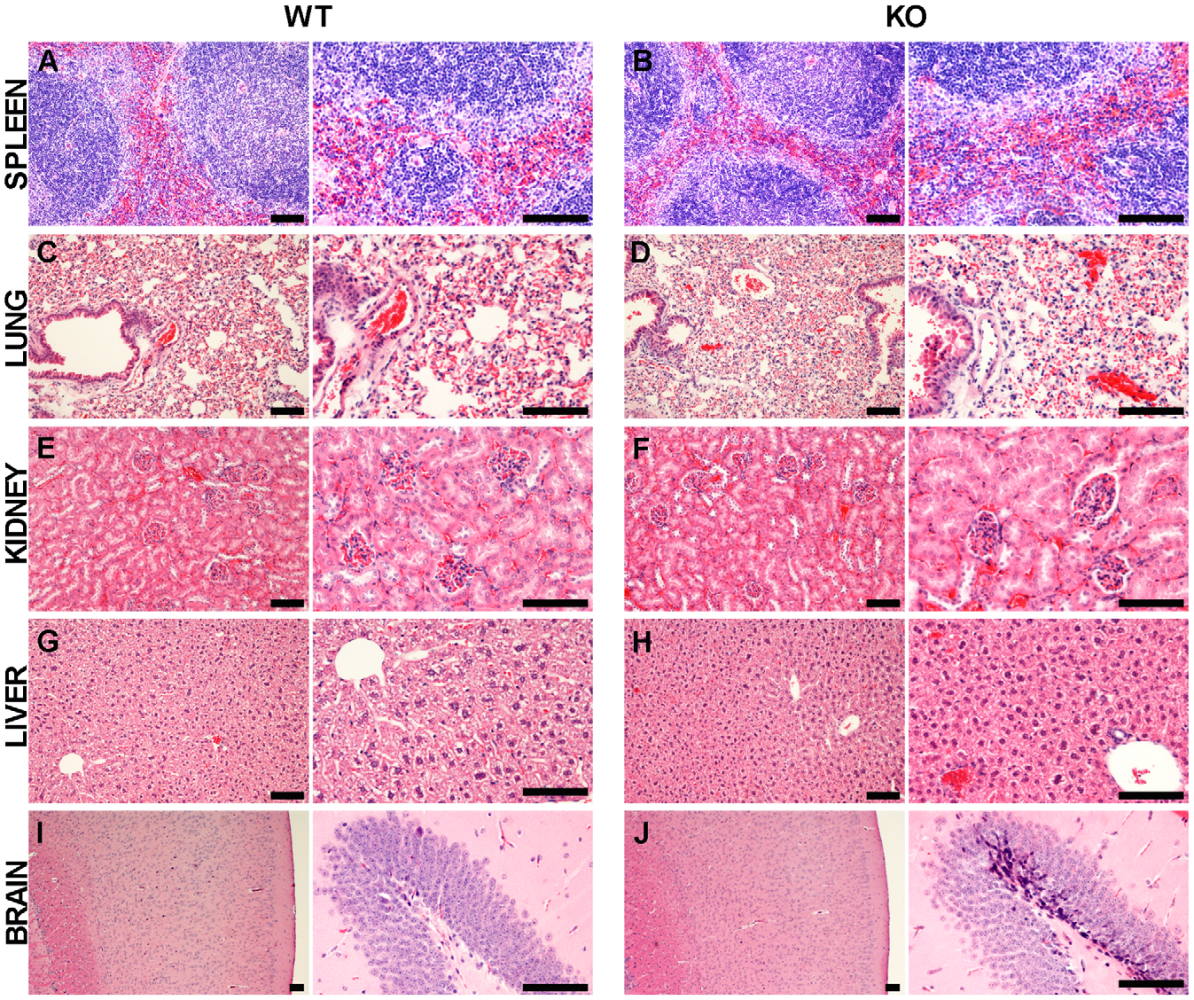
Comparison of tissue morphology in adult wild-type and twinfilin-2a knockout mice. Hematoxylin-eosin staining of paraffin-embedded sections of spleen (A, B), lung (C, D), kidney (E, F), liver (G, H), and brain (I, J) of 3-month-old wild type (WT) and knockout (KO) littermates. No morphological differences are evident between the tissues of wild-type and twinfilin-2a deficient mice. Scale bars represent 100 µm.

Twinfilin-2a knockout mice showed no obvious morphological or behavioural abnormalities, such as changes in general posture, size or activity, and they developed normally to adulthood and were fertile. Tissues of mutant mice were indistinguishable from those of wild-type littermates in size, weight and general appearance. To analyze the tissue morphology more closely, hematoxylin-eosin staining of tissues, i.e. spleen, lung, kidney, liver and brain, from three-month-old wild-type and knockout mice was performed (Figure 4A–J). Tissues were selected on the basis of where twinfilin-2a is most abundantly expressed in wild-type mice. Histological comparison revealed no obvious morphological differences between the tissues of wild-type and knockout mice.

**Figure 5:**
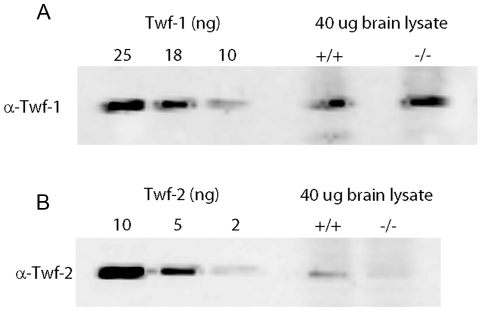
Relative amounts of twinfilin-1 and twinfilin-2a proteins in the brain lysates. Mouse cell extracts and indicated amounts of purified recombinant mouse twinfilin-1 (A) or twinfilin-2a (B) were run on polyacrylamide gels, and the proteins were visualized by Western blotting using isoform-specific twinfilin-1 (A) or twinfilin-2 (B) antibodies. Twinfilin-1 is 5–10 fold more abundant than twinfilin-2 in the brain lysate. Twinfilin-2 is almost non-detectable in the twinfilin-2a knockout brain lysate, suggesting that twinfilin-2a is the predominant twinfilin-2 isoform in the brain.

A plausible explanation for the lack of a strong phenotype in twinfilin-2a deficient mice could be a functional redundancy with other related protein(s). The best candidate for such a molecule is twinfilin-1. Although our qRT-PCR analysis did not reveal significant upregulation of twinfilin-1 expression in twinfilin-2a knockout mice, it is important to note that at least in the brain tissue twinfilin-1 is 5–10 fold more abundant at the protein level compared to twinfilin-2a (Figure 5). Thus, the total twinfilin levels in the brains of the twinfilin-2a knockout mice were decreased only by ∼10–20% compared to the wild-type mice.

## Discussion

Genetic studies on budding yeast and *Drosophila* revealed that twinfilin plays a central role in actin dynamics in lower eukaryotes [4], [16]. However, the function of twinfilin in vertebrate development and physiology has not been reported. Studies on vertebrate twinfilins are further complicated by the presence of three twinfilin isoforms with partially overlapping expression patterns and similar biochemical activities [12].

Here, we report that inactivation of one mouse twinfilin isoform, twinfilin-2a, does not result in gross abnormalities in mouse development or physiology. We speculate that the depletion of twinfilin-2a may be compensated by the presence of twinfilin-1, which is typically co-expressed in the same non-muscle tissues and cell-types with twinfilin-2a [11], [12]. Both proteins also interact with monomeric actin, filament barbed ends, heterodimeric capping protein, and PI(4,5)P_2_ in a similar manner [11], [12]. Similar functional redundancy has been previously reported also for isoforms of other central actin regulators such as Éna/VASP family proteins [22]–[25].

However, despite the biochemical similarities, the localizations of twinfilin-1 and twinfilin-2a in cells appear to be regulated through different pathways. Whereas twinfilin-1 localizes to the lamellipodial actin network and cell-cell contacts upon expression of dominant active Rac and Cdc42, respectively, these small GTPases do not appear to regulate the sub-cellular localization of twinfilin-2a [11]. It is therefore possible that twinfilin-2a knockout mice will display more subtle defects in physiological processes, which require specific regulation of the different twinfilin isoforms.

Previous studies have shown that both twinfilin-1 and twinfilin-2a contribute to endocytosis in cultured mammalian cells [7], [18]. Furthermore, twinfilin-2a was identified in an RNAi screen as a central protein promoting neuronal outgrowth [19]. Surprisingly, analysis of the knockout mice suggests that in intact animals the presence of twinfilin-2a is not required for central cell biological processes such as receptor-mediated endocytosis. Furthermore, the lack of detectable behavioral abnormalities or histological alterations in the brain of knockout mice does not support a central role for twinfilin-2a in neuronal outgrowth. One possible explanation for this discrepancy is that in animals the lack of twinfilin-2a in developing neurons could be compensated by elevated expression of twinfilin-1, whereas in the cell-culture model used by Yamada *et al.*, [19] twinfilin-1 over-expression would not be triggered. However, we did not detect significant up-regulation of twinfilin-1 mRNA or protein in any of the twinfilin-2a knockout mouse tissues tested.

In conclusion, our data show that twinfilin-2a is not an essential regulator of the actin cytoskeleton during development or in adult mice. However, twinfilin-2a might play a more subtle role during specific physiological processes that are not critical for the survival of mice in laboratory conditions. Considering the wide tissue distribution of twinfilin-1 and the reported functional similarities between twinfilin isoforms, we propose that the lack of twinfilin-2a is compensated by the presence of twinfilin-1. Thus, for analyzing the roles of twinfilin in mice, it will be necessary to generate double or triple knockouts of different twinfilin isoforms.

## Materials and Methods

### Isolation of mouse *twinfilin-2* genomic DNA

A PAC library was screened using twinfilin-2 cDNA as a probe. As a result six positive PACs were obtained. The presence of the exon-1 was tested by PCR using exon-1 forward primer MV33 and exon-2 reverse primer MV35. Two positive PACs were identified (491-J17 and 344-N17), and the PAC-DNA was isolated from these clones using Qiagen Large construct kit (Qiagen). PAC-DNAs were digested with EcoRV, EcoRI, BamHI, HindIII, BamHI+EcoRI and HindIII+EcoRV and the resulting fragments were separated on a 0,8% agarose gel in TAE buffer and blotted on to a Hybond N filters (Amersham). Filters were denatured in 0.5 M NaOH, 1.5 M NaCl, neutralized in 0.5 M Tris-HCl pH 8.0, 1.5 M NaCl and UV crosslinked. Prehybridization and hybridization was carried out at 65°C in Church buffer [22] with 0.2 mg/ml salmon sperm DNA. Filters were washed twice for 15 min in 0.1×SSC (15 mM sodiumchloride, 1.5 mM sodiumcitrate, pH 7.5) and 0.1% SDS at 65°C and exposed to an X-ray film. Hybridization with intron-1 as a probe gave positive bands of about 10 kb from EcoRV and EcoRI digested DNA. PAC-DNA was again digested with EcoRV and EcoRI, approximately 10 kb fragments were cut from the gel and cloned into pBSIIKS vector to the respective sites. Resulting colonies were screened with Colony lift Southern blotting using intron-1 as a probe with the same conditions as described before. One positive clone was isolated, 2B13. This 8.6 kb EcoRI-fragment was further tested by PCR for intron-1 and by restriction fragment analysis to confirm the presence of only one insert and subsequently sequenced. The genomic fragment contained exon 1 and exon 2 of the twinfilin-2 gene (Figure 1A).

### Generation of twinfilin-2a-deficient mice

To inactivate the *twinfilin-2* gene, a targeting construct with a neomycin resistance expression cassette containing a polyadenylation signal was inserted downstream of the ATG in exon 1. The genomic fragment 2B13 was digested with EcoRI+NcoI and the resulting piece containing the TATA box, transcription initiation site and exon 1 was cloned to pBSIIKS vector to give the left arm. Digestion of 2B13 with SpeI+XhoI and cloning the resulting piece containing intron 1 into pWH9 vector gave the right arm. The pWH9 vector contains the neomycin cassette. The cassette and the right arm were digested with XhoI and cloned to the unique XhoI site in the exon 1. The resulting targeting construct, containing a 5 kb left arm and 2.4 kb right arm was linearized with NotI digestion and electroporated into 129sv embryonic stem (ES) cells as described earlier [27]. The ES cells were subjected to G418 selection (350 µg/ml in ES cell medium) to obtain stable transfectants. To identify the clones with homologous recombination, EcoRI digested DNA was analyzed by Southern blotting using a 750 bp external probe and 800 bp internal probe. External probe identified a 8.6 kb fragment in the wild type and a 3.5 kb fragment in the mutant alleles. Internal probe identified a a 8.6 kb fragment in the wild type and a 11 kb fragment in the mutant alleles and nothing in the wild type. Three correctly targeted clones were injected into C57BL/6 blastocysts and the injected blastocysts were transferred into pseudopregnant foster mothers to generate chimeric mice. Resulting chimeric mice were mated with C57BL/6 mice and the germ line transmission of the mutant allele was tested by Southern blot analysis with the external and internal probes. For routine genotyping PCR reactions were designed with one pair of primers giving a product from the intron 1 and one primer pair with the sense primer in the neomycin cassette and the antisense primer in the intron 1.

### RNA extraction

Total RNA was isolated from various tissues of adult mice by TRIzol (Invitrogen) according to the manufacturer's protocol. Briefly, tissue samples were homogenized in a 15 ml tube with Polytron homogenizer (Glen Mills) in 1.0 ml of TRIzol per 100 mg of tissue. Homogenates were incubated for 10 min at room temperature and centrifuged at 12,000×g for 15 min at 4°C to pellet insoluble material and high-molecular-weight DNA. Chloroform was added, tubes were shaken for 15 seconds, incubated 3 min at room temperature and centrifuged at 12,000×g for 15 min at 4°C. After phase separation, RNA was precipitated with isopropanol, washed with 75% ethanol and resuspended in an appropriate volume of DEPC-treated water. RNA purity and quantity was ascertained from optical density at 260 and 280 nm. The samples were stored at −80°C until use.

### Nothern blot hybridization

For Northern blot analysis 5 µg of RNA was fractionated on a 1,2% denaturing agarose gel and blotted on to a Hybond N membrane (Amersham). The filters were UV-linked and hybridized with 32P-labeled cDNA probes specific for mouse twinfilin-2 or GAPD (glyceraldehyde phosphodehydrogenase) gene according to the standard protocol in Church buffer [26] at 65°C. Filters were washed twice in 0.2×SCC/1% SDS at 65°C and exposed to an x-ray film for 24 or 72 h at −80°C.

### PCR

Three micrograms of total RNA was reverse-transcribed at 42°C for 1 h in 20 µl solution containing 200 units of SuperScript II Reverse Transcriptase (Invitrogen) and 500 ng of Oligo(dT). The resulting cDNA was either immediately used for non-quantitative PCR or quantitative PCR (Q-PCR) or stored at −20°C until use. Non-quantitative PCR was performed in a final volume of 50 µl and containing 2 µl of the cDNA obtained by reverse transcription. PCR was performed with the following conditions: initial incubation 95°C for 3 min and 35 cycles at 95°C for 60 s, 60°C for 60 s and 72°C for 10 min. GAPDH was amplified as a control. The following oligonucleotides designed from the cDNA of twinfilin-2a, twinfilin-2b and GAPDH were used: ES79; CAGGACCAAGAGGAGAACTC and ES81; AATCAAGTCGGAAGAGGAGG for twinfilin-2a, ES80; ACTCTGCCTGCTTGCTCACC and ES81 for twinfilin-2b and ES126; GCAAAGTGGAGATTGTTGCCAT and ES127; CCTTGACTGTGCCGTTGAATTT for GAPDH. Q-PCR was applied to determine the expression levels of twinfilin-1 and twinfilin-2b in different tissues and was performed as described earlier [12]. GAPDH was amplified as a control.

### Western blotting

Tissue samples were dissected and either used directly or after storage at −80°C. Samples were briefly homogenized with a Polytron homogenizer (Glen Mills) in 10 volumes of ice-cold lysis buffer (50 mM TrisHCl, pH 7.4, 150 mM NaCl, 5 mM EDTA, 0.1% SDS, 1% deoxycholate, 1% Triton-X 100 and the complete protease inhibitor mixture (Roche). After 10 min extraction in the cold, tissue residues were removed by centrifugation for 20 min at 14,000 g at 4°C. Twenty micrograms of protein was applied per lane for SDS polyacrylamide gel electrophoresis analysis [28] on 12% gels, followed by electrophoretic transfer to a nitrocellulose membrane. Proteins were detected with affinity-purified and cross-reacted polyclonal antibodies against mouse twinfilin-1 and twinfilin-2 or mouse monoclonal antibodies against actin or GAPDH. Detection was performed with the appropriate secondary antibodies coupled to horseradish peroxidase and the ECL (enhanced bioluminescence) kit (Amersham). The Western blot analysis for examining the relative amounts of twinfilin-1 and twinfilin-2a/b proteins in the brain lysates was carried out as described in [11].

### Histochemistry

Paraffin sections were used for histological preparations. Tissues from 3 month-old knockout and wild-type littermates were fixed in 10% paraformaldehyde overnight at 4°C. Automated dehydration of tissues and subsequent paraffin embedding was performed with a Leica ASP 300S (Leica) device. Paraffin blocks, prepared with a Tissue-Tek device (Sakura), were cut and transferred to a Tissue-Tek Prisma/Film (Sakura) automated slide stainer and coverslipper for hematoxylin-eosin staining and mounting.
